# Robust Grape Cluster Detection in a Vineyard by Combining the AdaBoost Framework and Multiple Color Components

**DOI:** 10.3390/s16122098

**Published:** 2016-12-10

**Authors:** Lufeng Luo, Yunchao Tang, Xiangjun Zou, Chenglin Wang, Po Zhang, Wenxian Feng

**Affiliations:** 1 Key Laboratory of Key Technology on Agricultural Machine and Equipment, Ministry of Education, South China Agricultural University, Guangzhou 510642, China; luolufeng@tute.edu.cn (L.L.); 2005209012@stu.scau.edu.cn (C.W.); zhangpo@tute.edu.cn (P.Z.); 2 College of Mechanical Engineering, Tianjin University of Technology and Education, Tianjin 300222, China; 3 School of Civil and Transportation Engineering, Guangdong University of Technology, Guangzhou 510006, China; wenxianfeng0908@gdut.edu.cn

**Keywords:** grape detection, AdaBoost classifier, color components, harvesting robot

## Abstract

The automatic fruit detection and precision picking in unstructured environments was always a difficult and frontline problem in the harvesting robots field. To realize the accurate identification of grape clusters in a vineyard, an approach for the automatic detection of ripe grape by combining the AdaBoost framework and multiple color components was developed by using a simple vision sensor. This approach mainly included three steps: (1) the dataset of classifier training samples was obtained by capturing the images from grape planting scenes using a color digital camera, extracting the effective color components for grape clusters, and then constructing the corresponding linear classification models using the threshold method; (2) based on these linear models and the dataset, a strong classifier was constructed by using the AdaBoost framework; and (3) all the pixels of the captured images were classified by the strong classifier, the noise was eliminated by the region threshold method and morphological filtering, and the grape clusters were finally marked using the enclosing rectangle method. Nine hundred testing samples were used to verify the constructed strong classifier, and the classification accuracy reached up to 96.56%, higher than other linear classification models. Moreover, 200 images captured under three different illuminations in the vineyard were selected as the testing images on which the proposed approach was applied, and the average detection rate was as high as 93.74%. The experimental results show that the approach can partly restrain the influence of the complex background such as the weather condition, leaves and changing illumination.

## 1. Introduction

Grapes are one of the common fruits in the world. Grape yields are increasing along with the development of the grape industry. However, grape harvesting is a time-consuming and labour-intensive procedure [1], therefore it is very necessary to develop an automatic grape harvesting system. For automatic harvesting, the major task is to detect and locate the grape clusters in a vineyard by using artificial vision. This is the heart of any grape harvesting robot. However, the dynamic and complex vineyard environment makes the target detection difficult. It is not an easy job to develop a vision system as intelligent as a human who can easily recognize grape clusters in a vineyard, especially when the color of the grapes and the background is similar.

In recent decades, scholars from different parts of the world have proposed a large number of target detection methods for harvesting robots, but so far, an ideal method to completely solve the difficulties posed by the complex vineyard environment has yet to be found. To overcome the influences caused by uneven illumination conditions and color similarities, various vision sensors have been used for detecting the fruits [2]. In early years, Kondon et al. [3] used the spectral characteristics of grapes to identify and locate the harvesting target. Jimenez et al. [4] adopted a laser-based computer vision system for fruit detection, which is based on an infrared laser range-finder sensor that provides range and reflectance images. Wang et al. [5] and Font et al. [6] used a binocular stereo vision to recognize and locate fruits in an unstructured environment with varying illumination. To detect apples in the canopy, Feng et al. [7] designed a machine vision system consisting of a time-of-flight (ToF) camera and a digital color charge coupled device (CCD) camera. The detection methods based on the advanced vision sensors and fusion of multiple image data can achieve higher correct detection rates. However, the high-cost of the sensors is the key shortcoming for commercial application [8].

In the aspect of fruit detection in images, Berenstein et al. [9] utilized the difference in edge distribution between the grape clusters and the foliage to detect the grape clusters for an automatic selective vineyard sprayer. In [10], the number of grape berries were counted by detecting specular spherical reflection peaks in RGB images captured at night under artificial illumination. In [11], a detection approach for the grapes in the vineyard images captured at night was proposed by defining a region of pixel intensities in the RGB color space based on a trial and error procedure. Luo et al. [12] proposed an image segmentation approach for grape in the vineyard based on the improved fuzzy clustering method by artificial swarm and the H-I color component, its accuracy was 90.33%. In [13] recognized the kiwifruit at nighttime by extracting R-G color components. The success rate of the recognition algorithm was 88.3%. In [14], a best color segmentation method for the grapes in the vineyard images captured at night was developed by extracting the H component of the HSV color space to estimate vineyard yield. In [15], the conventional RGB color images were transformed into the YIQ color space, and then the threshold of color intensity was obtained by a trial and error manual operation. In [16], the threshold of color intensity was obtained by analyzing the histogram of the transformed images. To detect the red peaches in orchard images, a classification method was proposed in [17] based on different linear color models in the RGB vector color space, and then the segmentation of the pixels of the image was performed by the classifier. In [18], a grape bunch segmentation algorithm combining color and texture information and the use of a support vector machine(SVM) was proposed, one new filter and one new feature were designed for eliminating false detected bunches. In [19], a grape berry detection method based on both visual texture and shape was developed, which could detect the green berries against a green leaf background.

From the above fruit detection approaches we can know that most of current studies on grape detection in the vineyard have focused on the pixel-based color segmentation and the texture-based detection approaches, whose main goals were to estimate the vineyard yield [10,18,19,20] or to identify the targets for automatic selective vineyard sprayer [9]. These approaches relied more on some effective color components and the descriptor of the texture. However, in actual cases, selecting the best color component was still one of the difficulties in color image segmentation [21]. Moreover, it was also difficult to identify a target only by relying on one kind of color space classification in the dynamic and complex vineyard environment.

With the rapid development of machine learning technology, more and more vision learning approaches have started to be used for detecting fruits in outdoor scenes [22]. Chamelat et al. [23] developed a grape detection approach based on support vector machine (SVM) by selecting Zernike moments and color information as features. In [8], an algorithm to detect tomatoes in greenhouse scenes was proposed, in which the possible tomato objects were firstly identified by extracting the Haar-like features of grey scale image and classifying by the AdaBoost classifier, and then the false classification were eliminated by color analysis approach. In [24], a two-step coarse-to-fine wheat ear detection mechanism was proposed. To improve the detection accuracy, the machine learning technology was used to identify candidate targets in the coarse-detection step. In [25], a pedestrian detection approach was designed by establishing coupled strong classifiers based on AdaBoost framework. To predict the class of berry color, in [26], the mean of the RGB values of all grape berries detected in one image were used for statistical analysis using linear discriminant analysis.

Inspired by the machine learning methods and the pixel-based color segmentation methods, this paper focused mainly on the combination of two methods to find a robust detection approach for the grape harvesting robots. In the vineyard, the environments conditions are variable, so the robustness of unsupervised machine learning methods may be at risk [27]. Therefore supervised machine learning methods are of special interest in this field. Since the training set of the supervised algorithm is prepared a priori, which establishes what features will correspond to the elements of a class, this reduces the uncertainty of the complex vineyard [20]. In the supervised machining learning methods, one of the most successful paradigms is ensemble learning [28], and AdaBoost [29] is one of the most influential ensemble methods. In contrast to ordinary machine learning approaches which try to generate one learner from training data, AdaBoost methods try to construct a set of base learners and combine them.

To eliminate the influences caused by complex environments and improve the detection rate of the grape clusters in the vineyard, a new vision sensing algorithm fused the advantages of multiple color space (e.g., RGB, HSV, La*b*) based on the Adaboost framework was developed in this paper. Firstly, the training data was prepared by manually cropping images captured in the vineyard. Several effective color components that can distinguish well the grape clusters from the background were extracted from multiple color space by contrasting the background and grape clusters, and then the corresponding linear classification models were constructed based on these components. Secondly, a strong classifier was generated by using the new concept that assembled multiple linear classification models based on Adaboost framework. Thirdly, all of the pixels of the conventional vineyard color images were classified into grapes or background by using the strong classifiers. Finally, the morphological noise was eliminated by the region threshold method and opening-and-closing operation, and the grape clusters were marked finally by using the enclosing rectangle method.

## 2. Materials and Methods

### 2.1. Image Acquisition and the Dataset

**Figure 1:**
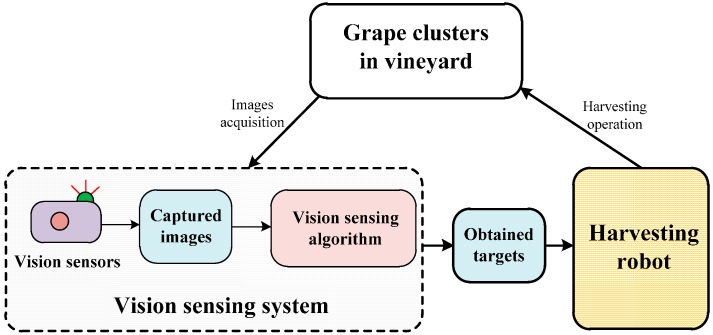
The vision sensing system of the harvesting robot.

The vision sensing system of the harvesting robot included vision sensors and sensing algorithm, which can be simply shown in Figure 1. The vision sensors (e.g., cameras) were mainly responsible for collecting vineyard images, and the sensing algorithms were responsible for detecting and extracting the harvesting targets from the collected images.

For developing and testing the developed algorithm, images of grape clusters in the vineyard were acquired in late July 2015 which was the harvesting season for the most common cultivar ‘Summer Black’ at the Chadian Grape Science Park, Tianjin, China. There are greenhouse and outdoor grapevine trellis systems in the park. The greenhouse grapevine trellis system adopted 3-m-high T-shaped brackets as the supporting frame, and the size of row spacing interval of grapevines was about 3 m. The outdoor grapevine trellis system used 1.8-m-high Y-shaped brackets as the supporting frame, and the spacing interval of grapevines was about 2.5 m. A total of 200 ripe grape cluster images were captured using a color digital camera (D5200, Nikon, Wuxi, China) with a resolution of 3872 × 2592 pixels. The auto-exposure control mode based on the shutter speed priority was adopted, and the exposure time was fixed to 1/100 s. The photograph distance was approximately 600–1000 mm, which was determined according to the best working distance between the grape clusters and the harvesting robot in the vineyards [1]. Among the 200 images, 60 images were collected in sunny frontlight, 60 images were collected in sunny overshadow, and the remaining 80 images were collected in overcast lighting conditions. To improve the image processing speed, all images were resized to 600 × 800 pixels using the bicubic interpolation algorithm.

**Figure 2:**
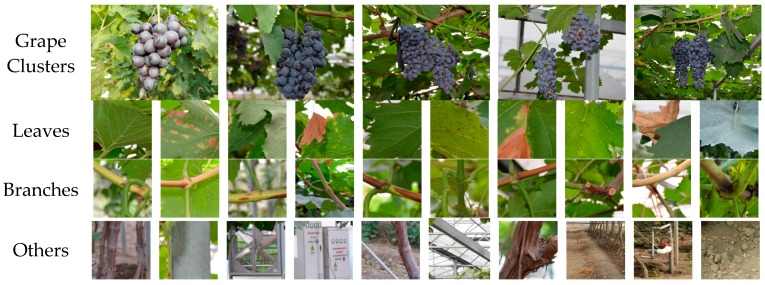
Examples of the dataset images. The **top** row listed the images of grape clusters captured under different lighting conditions, and the **lower** rows were background including leaves, branches, and other obstacles in the vineyard.

To train the classifiers constructed in this study, 80 images were randomly selected from the 200 images captured under different lighting conditions, from which 400 grape samples and 800 background samples were manually cropped as the training dataset. In addition, 60 images were randomly selected from the remaining 120 images, and 300 grapes and 600 background samples were cropped from these images as the testing dataset. To decrease the influence caused by the noise, the pixels window of each sample was set to 7 × 7 pixels, and the average pixels value of each windows were viewed as a sample data. The background samples contained leaves, branches and other objects, and all samples were labeled separately, 1 for grape and −1 for background. Examples of the dataset images are shown in Figure 2.

### 2.2. Detection Algorithm of Grape Clusters

**Figure 3:**
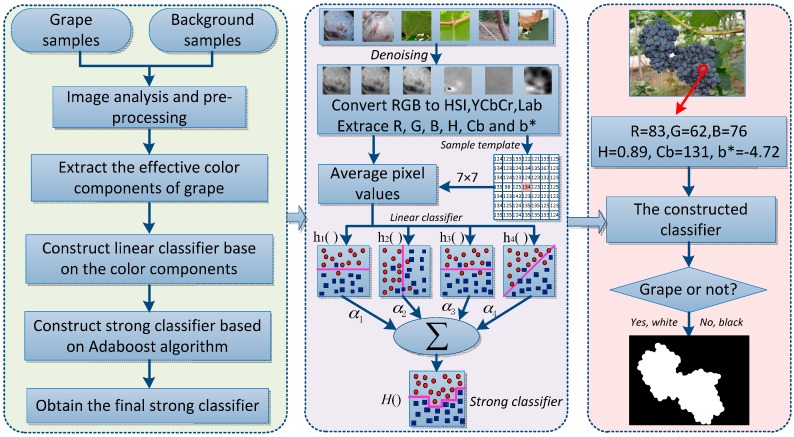
Principle of the proposed approach.

Principle diagram of the proposed grape detection algorithm is shown in Figure 3, which can be encapsulated in following steps: firstly, the images captured at vineyard were pre-processed, and several effective color components (e.g., H, Cb, b*, R and B) were extracted by contrasting and analyzing the color difference between the background and the grape clusters. Secondly, four linear classification models were constructed based on the dataset and these extracted components. Thirdly, the strong classifier was obtained by training the four weaker classifiers using the AdaBoost framework, which could be calculated by the sum of four weak classifiers multiplying their weights. Finally, the pixels of the captured images were distinguished and then identified by using the strong classifier. If the pixel it was grape then was set 1 (white), or set 0 (black). The noises in the binary images were eliminated by the region threshold method and morphological filtering, and the grape clusters were marked finally by using the enclosing rectangle method.

#### 2.2.1. Color Analysis and Extraction of Multiple Effective Color Components

**Figure 4:**
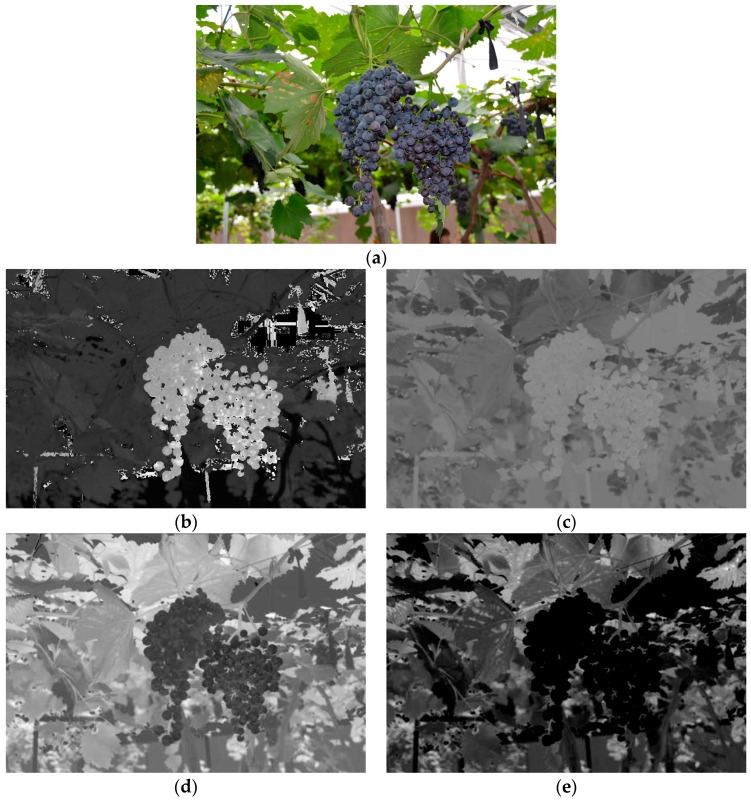
Several effective color components of images which can distinguish well the grape clusters from the background. (**a**) Original image; (**b**) H component image; (**c**) Cb component image; (**d**) b* component image; and (**e**) R-B component image.

**Figure 5:**
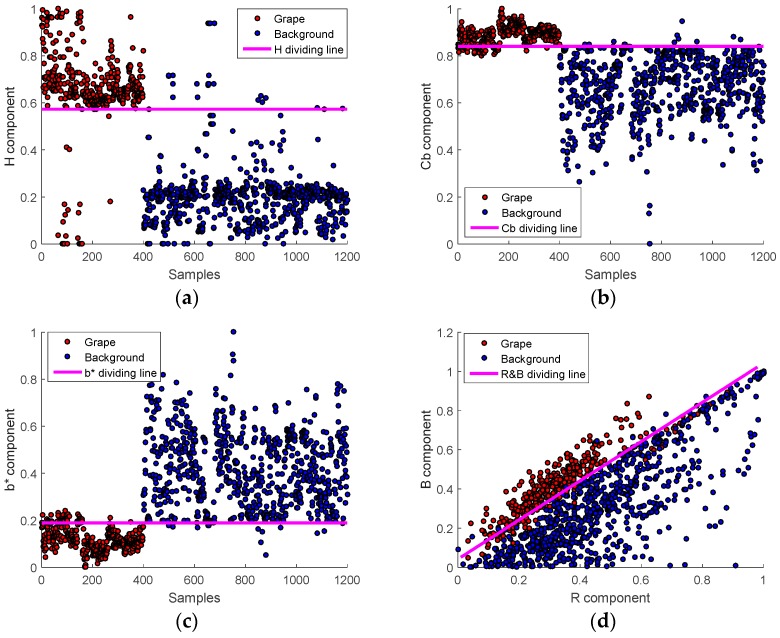
The distribution of average pixel value of 1200 samples on H, Cb, b*, B&R components and the corresponding dividing lines between grape and background (**a**) the dividing line H = 0.5736 and its *LCE* is 6.75%; (**b**) the dividing line Cb = 0.8405 and its *LCE* is 8.5%; (**c**) the dividing line b* = 0.1889 and its *LCE* is 12.08%; and (**d**) the dividing line B = 1.2 × G − 0.05 and its *LCE* is 10.83%.

The main methods used to recognize the fruits in the images were shape-based analysis and color-based analysis [30]. Considering the irregular contours of the grape clusters, color-based analysis was adopted in this study. The color was combined of red (R), green (G) and blue (B) which was usually called three primary colors [31]. From the three primary colors, different kinds of color spaces can be calculated by using either linear or nonlinear transformations [21], such as HSI, YCbCr and L*a*b* etc. To find the more effective color components for the summer black grape, in the preliminary works of this study, the grape images captured in the vineyard conditions were observed by decomposing them to various color spaces [8]. The detailed steps are as follows: firstly, because a substantial amount of noise that caused by varying illumination in the vineyard usually existed in the captured images, the mean filter was implemented to minimize the disruption to target detection. Secondly, the images were transformed from RGB to YCbCr, HSI, L*a*b, and then the color components of R, G, B, Y, Cb, Cr, H, S, I, L*, a*, and b* were extracted by using the Matlab software, respectively. Thirdly, the color difference between the grape clusters and background (e.g., the leaves, branches, sky and so on) were contrasted and analysed by the visual and quantitative evaluating methods which could be seen from the Figure 4 and Figure 5. It could be found that the H, Cb, b* and R-B components were the more effective color components that could preferably separate the summer black grapes from the background. Therefore, the several color components were elected as the candidate color components to construct the corresponding simple linear classifiers. The H component was computed by Equation (1) [32]:

(1) $$H=\{\begin{array}{cc} \theta & if\;B\leq G\\ 360-\theta & \mathrm{otherwise} \end{array}$$

where R, G and B were the three primary colors, and $\theta =\arccos\left\{\frac{0.5\times \left[\left(R-G\right)+\left(R-B\right)\right]}{{\left[{\left(R-G\right)}^{2}+\left(R-B\right)\left(G-B\right)\right]}^{1/2}}\right\}$

The Cb component was computed by Equation (2) [32]:

(2) Cb=(−0.1687×R−0.3313×G+0.500×B)+128

The b* component could be computed by Equation (3) [33]:

(3) $$b^{*}=200\left[f\left(Y\right)-f\left(\frac{Z}{1.192}\right)\right]$$

where $f\left(t\right)=\{\begin{array}{cc} t^{1/3} & t>0.008856\\ 7.787t+0.138 & t\leq 0.008856 \end{array}$, in which $\left[\begin{array}{c} X\\ Y\\ Z \end{array}\right]=\frac{1}{0.17697}\left[\begin{array}{ccc} 0.49 & 0.31 & 0.20\\ 0.17697 & 0.8124 & 0.01063\\ 0.00 & 0.01 & 0.99 \end{array}\right]\left[\begin{array}{c} R\\ G\\ B \end{array}\right]$.

#### 2.2.2. Construct Linear Classification Models Based on the Extracted Color Components

To separate the grape clusters from background, four linear classification models were firstly established based on the extracted color components (H, Cb, b* and G&R). Among the four classification models, the H, Cb and b* classification models only relied on single channel, and the B&G classification models was related to two channels. These models could be thought as a simple threshold or linear operation on the color components value. To get the linear equations of these classification models more easily, we set $\left(x_{1},x_{2},\ldots x_{N}\right)$ as the training sample sets, in which $x_{i}$ denoted the ith training sample and N is the total number of samples. Then, the distribution of 1200 samples of pixel value on H, Cb, b*, B&G components were plotted to look for the ideal linear classification models, which was shown in Figure 5, the red dots represents grape and the blue dots represents background. The dividing lines equations of these classification models are uniformly defined by the euqation $L_{c}\left(x_{i},p\right)=0$, in which c was the candidate color components, p were the threshold or equation parameters. By combining the threshold and linear classification principles, these dividing lines could be described by Equation (4):

(4) $$\{\begin{array}{c} L_{H}\left(x_{i},\;H_{th}\right)=H\left(x_{i}\right)-H_{th}\\ L_{\mathrm{Cb}}\left(x_{i},Cb_{th}\right)=Cb\left(x_{i}\right)-Cb_{th}\\ L_{b*}\left(x_{i},La{b_{b}}_{th}\right)=La{b_{b}}_{th}-Lab_{b\left(x_{i}\right)}\\ L_{B\&R}\left(x_{i},a,b,c\right)=a*B\left(x_{i}\right)+b*R\left(x_{i}\right)+c \end{array}$$

In which the parameters of $H_{th}$, $Cb_{th}$, and $Lab\_b_{th}$ indicated the optimal classification threshold of H, Cb and b*, respectively. $H\left(x_{i}\right)$, $Cb\left(x_{i}\right)$, $Lab\_b\left(x_{i}\right)$, $B\left(x_{i}\right)$ and $G\left(x_{i}\right)$ were the specialized color component values of the sample $x_{i}$. a, b and c were the parameters of the line equation of the *B*&*R* classifier.

Training the parameters in the Equation (4) was a very critical step for the linear classification models to separate the grape from background. To get the optimal parameters, the lowest classification error (*LCE*) method was employed in this study. The optimal parameters were searched by iterating the parameters among the range of the predefined value, which could minimize the error rate of the classification models. The *LCE* method could be described by Equation (5):

(5) $$LCE=arg\;min\left\{\overset{N}{\underset{i=1}{\sum }}T\left(x_{i}\right)\right\}/N$$

in which $T\left(x_{i}\right)$ was the sign function. When the $x_{i}$ was misclassified by the specialized linear classification model, its value was 1, otherwise was 0.

Figure 5a–d show the distribution of average pixel value of 1200 samples on H, Cb, b*, B&R components and the corresponding dividing lines between grape and background were obtained by the *LCE* method.

#### 2.2.3. Construct Strong Classifier Based on the AdaBoost Framework

The detection method and model are the key technology to precisely identify the grape clusters. Although these classification models constructed in Section 2.2.2 can be used to identify grape object, most of these models play a limited role in grape clusters detection. To regenerate a strong classifier, a new classification application was investigated by combining the AdaBoost algorithm and the various constructed linear classifiers. The AdaBoost algorithm is an iterative algorithm which was proposed by Freund and Schapire [34,35] according to the online assignment algorithm. In the AdaBoost algorithm, a different set of training samples is obtained by changing the distribution of the sample weight [36,37]. At the beginning, the weight of every sample was set at the same value, and then the first weak classifier was trained according to the given weight. After a round of iterative, the corresponding weight for the misclassified samples would be increased, and the weight for the correct classification sample would be reduced, so the misclassified samples were highlighted, a new weight for every sample were regenerated. The second weak classifier was trained using the new weight, and in a loop until the last weak classifier was trained out. Finally, a desired strong classifier was obtained by a weighted average of the multiple weak classifiers. In order to illustrate the proposed classifier clearly, based on the discussion in Section 2.2.2 and Equation (4), we described the weak classifiers $h_{t}\left(x_{i}\right)$ by Equation (6):

(6) $$h_{t}\left(x_{i}\right)=\{\begin{array}{cc} 1 & L_{t}\left(x_{i},p\right)\geq 0\\ -1 & otherwise \end{array}$$

where t was the series number of the weak classifiers and also was a running index which means the iterative times in the AdaBoost algorithm (1≤t≤T), $L_{t}\left(x_{i},p\right)$ was the dividing line of the corresponding classifier and p are the parameters set of the classifier. When the value of $L_{t}\left(x_{i},p\right)$ was greater than 0, $x_{i}$ was considered to be grape cluster (1), otherwise, $x_{i}$ was view as background (−1).

The pseudo code and the detailed training process of the proposed classifier are as follows (in Algorithm 1):
**Algorithm 1.** The pseudo code of the constructed classifier.**Input:** Training samples set $Data=\left(x_{1},x_{2},\ldots x_{N}\right)$ and number of learning rounds *T*.
1.$D_{1}\left(x_{i}\right)=1/m$ % Initialize the weight distribution for all samples2.**For** t=1, 2, …*T*  $h_{t}\left(x_{i}\right)=LCE\left(Data,D_{t}\left(x_{i}\right)\right)$ % Train a learner $h_{t}\left(x_{i}\right)$ from Data using $D_{t}\left(x_{i}\right)$ based on the LCE method3.  $\varepsilon_{t}=\sum_{i=1}^{N}D_{t}\left(x_{i}\right)\;\left[y_{i}\neq h_{t}\left(x_{i}\right)\right]$ % Calculate the error of $h_{t}\left(x_{i}\right)$4.  **If** $\varepsilon_{t}$ > 0.5 **then break**5.  $\alpha_{t}=\frac{1}{2}log\left[\frac{\left(1-\varepsilon_{t}\right)}{\varepsilon_{t}}\right]$ % Determine the weight of $h_{t}\left(x_{i}\right)$6.  $D_{t+1}=\frac{D_{t}\left(x_{i}\right)}{Z_{t}}\times \{\begin{array}{cc} e^{-\alpha_{t}} & if\;y_{i}=h_{t}\left(x_{i}\right)\\ e^{\alpha_{t}} & otherwise \end{array}$ % Update the weight distribution for all samples7.**End****Output:** The proposed classifier $H\left(x_{i}\right)=sign\left(\sum_{t=1}^{T}\alpha_{t}h_{t}\left(x_{i}\right)\right)$

Firstly, allocated an initial weight for each training sample, $D_{1}\left(x_{i}\right)=1/N$. Then, the first weak classifiers $h_{1}\left(x_{i}\right)$ from the training dataset using the weight $D_{1}\left(x_{i}\right)$ are trained. The training step searched for the most suitable classifier $L_{t}\left(x_{i},p\right)$ and its corresponding parameter p that could minimize the misclassified error $\varepsilon_{t}$ by using the *LCE* method. The $\varepsilon_{t}$ could be calculated by Equation (7):

(7) $$\varepsilon_{t}=\overset{N}{\underset{i=1}{\sum }}D_{t}\left(x_{i}\right)\;\left[y_{i}\neq h_{t}\left(x_{i}\right)\right]$$

where $\varepsilon_{t}$ is the sum of weights of samples which were misclassified. $y_{i}$ is the label value of the *i*th sample which had been set as 1 or −1, $D_{t}\left(x_{i}\right)$ is the weight of the *i*th sample, and N is the total number of sample. If $\varepsilon_{t}$ is greater than 0.5, the program will come out the round of iteration.

After obtaining the weak classifier $h_{t}\left(x_{i}\right)$, the weight of the trained weak classifier is determined by the Equation (8):

(8) $$\alpha_{t}=\frac{1}{2}log\left[\frac{\left(1-\varepsilon_{t}\right)}{\varepsilon_{t}}\right]$$

After finishing the round of iteration, to train the following weak classifier [38], the weight of each sample is updated by Equation (9):

(9) $$D_{t+1}=\frac{D_{t}\left(x_{i}\right)}{Z_{t}}\times \{\begin{array}{cc} e^{-\alpha_{t}} & if\;y_{i}=h_{t}\left(x_{i}\right)\\ e^{\alpha_{t}} & otherwise \end{array}$$

where $Z_{t}$ is a normalization factor which enables $D_{t+1}$ to be distributed and calculated by Equation (10):

(10) $$Z_{t}=\overset{N}{\underset{j=1}{\sum }}D_{t}\left(x_{i}\right)$$

In practice, there is no one single weak classifier which can perform the classification task with low error. To solve this problem, a final strong classifier is constructed by combining these trained weak classifiers and multiplying their weights. The final strong classifier is described by Equation (11):

(11) $$H\left(x_{i}\right)=sign\left(\overset{T}{\underset{t=1}{\sum }}\alpha_{t}h_{t}\left(x_{i}\right)\right)$$

where $H\left(x_{i}\right)$ denotes the function of the final strong classifier, and sign(⋅) is a sign function. If the numerical value of $\sum_{t=1}^{T}\alpha_{t}h_{t}\left(x_{i}\right)$ is greater than 0, $\;H\left(x_{i}\right)$ is 1, otherwise, $H\left(x_{i}\right)$ is −1.

Finally, the proposed classifier is obtained by training the final strong classifier $H\left(x_{i}\right)$ using the 1200 samples. During the iterative training process, the errors were monitored to reflect the rate and efficiency of weak classifiers. Maximum iterations were restricted to 5000. When the classification error of the classifier was less than 0.05, or the iteration number was more than 5000 times, the iteration was terminated. Each round of iteration automatically produces an optimal weak classifier. The detail parameters of the trained classifiers were listed in Table 1.

#### 2.2.4. Images Pixels Classification and Targets Extraction

**Figure 6:**
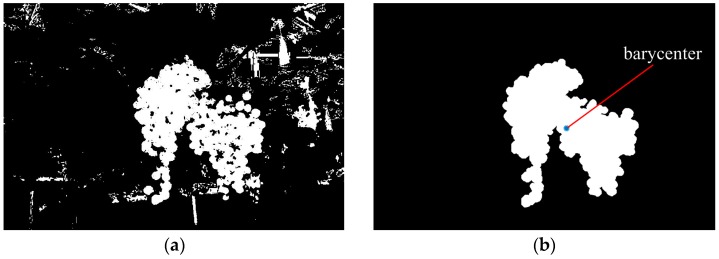

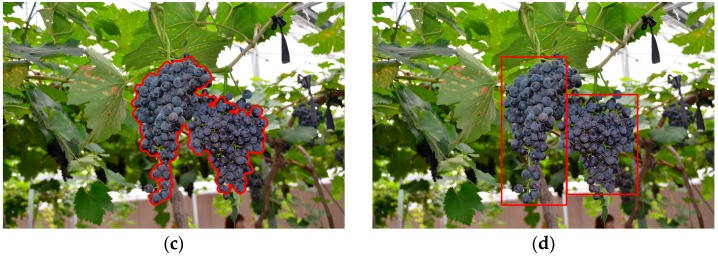
The detection processes of grape clusters in Figure 3a. (**a**) Obtained binary image by using the strong classifier; (**b**) Obtained grape region image after eliminating noises and executing a morphological operation (blue point was the barycenter); (**c**) The contour of the detected grape clusters; and (**d**) The enclosing rectangle were drew in the original image.

After finishing the training of the final strong classifier, we used it to distinguish the pixels of the captured images and identify if it is grapes or not. A sub-window (7 × 7 pixels) for every pixel was allocated in a captured image using a sliding window approach, and then calculated out the average pixel values of each sub-window in the images of the elected color components, and input the values into the strong classifier to verify whether the pixels were classified as grape. If the result of the final strong classifier was positive, the pixel would be considered to be grape (white), and if the result was negative, the pixel would be considered to be background (black). Figure 6a is the result image of Figure 4a obtained by using the strong classifier.

However, in an actual situation, there would still be a small amount of pixels whose colors are very similar to grapes that would not be correctly classified. Although the misclassified pixels were less than 10% of all pixels in most situations, they have a great influence on the correct detection. Therefore, the small discrete regions of the background pixels were removed by the region threshold method, which was based on finding the biggest region of neighboring white pixels in the image and eliminating all regions which were smaller than 1/10 of the biggest region [13]. In addition, for the adhesion noise, by choosing a radius of 5 of the disc structure element, an open filtering was implemented to discard these noises, and subsequently a close operator was executed to recover the contour of grape cluster. Finally, the morphological region was used to fill the hole in the image and obtained the pixel regions of the targets. The detection processes of the grape clusters in Figure 4a were shown in Figure 6. The pseudo code of image segmentation and targets extraction was as follows (in Algorithm 2):
**Algorithm 2.** The pseudo code of image segmentation and targets extraction.**Input:** Captured vineyard images**For all** pixels in the image  **If** (H(⋅)>0)  Pixel binary value=1  **Else**  Pixel binary value =0  **End****End****For all** pixel regions(value for 1) in the binary image  Eliminate the regions which are smaller than 1/4 of the biggest region  Perform open and close operator to delete the adhesion noise  Fill the hole for every pixel regions  Draw the enclosing rectangle for every pixel regions**End****Output:** Binary image that contains only the grape clusters

After obtaining the pixel regions of grape clusters, the targets were marked by using the enclosing rectangle method. In this method, the bounding box of the grape cluster was computed by the regionprops function of Matlab, and then the rectangle for the corresponding grape clusters was drawn. When there are two adjoining grape clusters, the barycenter of the pixel region of grape clusters was firstly calculated, and then the region was split into two parts by using the dividing line that passed through the barycenter from top to bottom, and the enclosing rectangle of each region was calculated, respectively. However, the detection of three or more adjoining and overlapping grape clusters was still a tricky problem, which has not been discussed in this study. Further research will be needed to solve that problem.

## 3. Experiments and Results

To validate the performance of the proposed algorithm, five experiments were performed. Firstly, the accuracy of the proposed classifiers was tested based on the 900 labeled testing samples to investigate its robustness. Secondly, 200 images captured at vineyard under different lighting conditions were selected as the testing images on which the proposed algorithm was applied. Thirdly, the proposed method’s robustness against adjacent and occlusion conditions were investigated and analyzed. Fourthly, the performance of the proposed algorithm was compared with other a bunch detection methods from the aforementioned literature. Finally, the interactive performance of the proposed algorithm was investigated. In order to facilitate the analysis of the experimental data, the false negative rate (FNR), false positive rate (FPR) and true positive rate (TPR) were used to evaluate the result of experiments, which were defined by Equations (12)–(14). All experiments of the algorithm developed in this study were performed on Matlab(R2015a) with an Intel (R)Core(TM)i5-3230M CPU@2.60 GHz:

(12) $$\mathrm{FNR}=\frac{\mathrm{False}\;\mathrm{negative}}{\mathrm{False}\;\mathrm{negative}+\mathrm{Correctly}\;\mathrm{done}}\times 100\%$$

(13) $$\mathrm{FPR}=\frac{\mathrm{Missed}\;\mathrm{done}}{\mathrm{Total}\;\mathrm{count}}\times 100\%$$

(14) $$\mathrm{TPR}=\frac{\mathrm{Correctly}\;\mathrm{done}}{\mathrm{Total}\;\mathrm{count}}\times 100\%$$

### 3.1. Accuracy of the Developed Classifier

In order to verify the accuracy of the proposed classifier, the experiment was conducted on the 900 testing samples, which included 300 grape samples and 600 background samples. The classification results of the proposed classifier were compared with the weak linear classifiers, and the experimental results were analyzed and counted by the true positive rate (TPR) and the false positive rate (FPR). A detailed comparison of the classification results is shown in Table 2.

From the table we can see that the proposed classifier could achieve the highest accuracy, which reached up to 96.56%. In the weak classifiers, the total accuracy of the weak classifier $h_{1}\left(x_{i}\right)$ was the highest, reaching 93.67%, and the weak classifier $h_{3}\left(x_{i}\right)$ had the lowest, only 81.33%. The classification accuracy of all weak classifiers was lower than the strong classifier. By analyzing the results, it can be seen that the proposed classifier based on the AdaBoost framework improved the efficiency and accuracy of the classification, therefore it could be well used to identify the grape clusters from the complex background.

### 3.2. Detection of the Grape Clusters under Different Lighting Conditions

**Figure 7:**
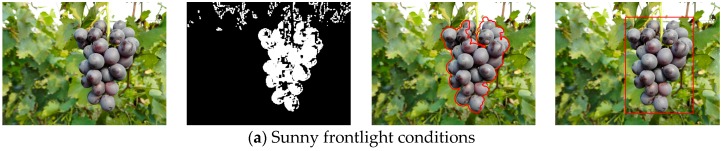

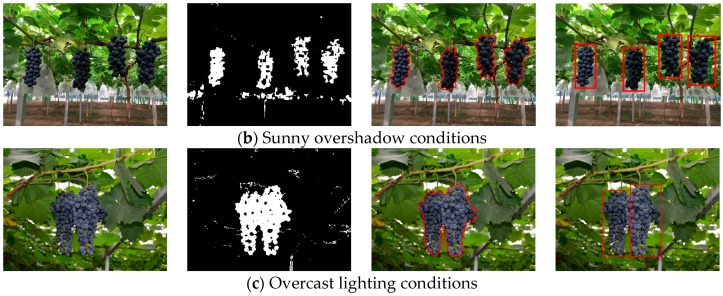
The detection results of the grape clusters from the images captured under three different lighting conditions by the developed method. (**a**) Image captured under sunny frontlight conditions; (**b**) Image captured under sunny overshadow conditions; (**c**) Image captured under overcast lighting conditions.

In order to investigate the performance of the detection algorithm under varying lighting conditions and complex backgrounds, 200 summer black grape images that were captured from different perspectives at the vineyard were tested using the developed detection algorithm in this paper. Among the 200 images, 60 images were collected under sunny frontlight conditions in which 136 grape clusters could be found, 60 images were collected under sunny overshadow conditions in which 129 grape clusters could be found, and the remaining 80 images were collected under overcast lighting conditions in which 182 grape clusters could be found. The tests were performed under above three different lighting conditions, respectively. The segmentation and detection results of one of the three conditions are shown in Figure 7. From the figure we can see that the grape clusters in original images were correctly identified and the backgrounds (green leaves, surface and sky, etc.) can be well classified. However, some iron frames, petioles, stipes and dead leaves can’t be identified at times, and a large part of the pixels were wrongly classified as grape, which can be seen in Figure 7b. However, these pixel points could be effectively removed after the denoising process, and the final detection effects were better.

In order to quantitatively evaluate the performance of the developed detection approach the false negative rate (FNR), false positive rate (FPR) and true positive rate (TPR) of the experiment were analyzed. The total of false negatives (where the background was misclassified as grape clusters) and missed grape clusters were manually counted. Table 3 lists the FNR, FPR and TPR of the proposed method under three different lighting conditions. In this developed detection approach, 93.74% of the actual numbers of the grape clusters were correctly identified. The false negative rate was about 4.34%. About 6.26% of the grape clusters were not detected. The true positives rate was the highest under the overcast lighting and reached to 95.05%.

### 3.3. Performance of the Proposed Method against Adjacent and Occlusion Conditions

**Figure 8:**
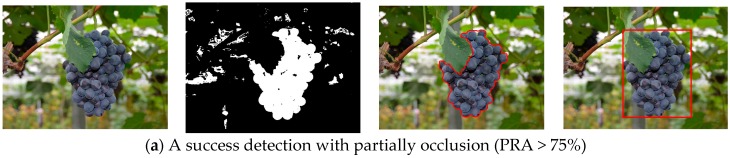

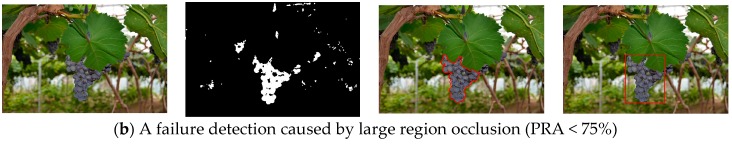
Detection process of the grape clusters under the different occlusion degrees. (**a**) a success detection with partially occlusion (PRA > 75%); and (**b**) a failure detection caused by large region occlusion (PRA < 75%).

In the detection experiment, grape clusters that were adjacent or partially occluded were tested. In these situations, when the grape clusters were not greatly covered by other obstacles, the grape clusters could still be well detected using the developed approach. For example, Figure 7c shows that the grape clusters were correctly detected although two grape clusters in the image are adjacent. Figure 8a shows that the grape cluster was accurately detected although the upper-left part of the grape cluster was occluded by leaves. However, if the grape clusters were largely hidden by foliage, the recognition accuracy of the grape clusters would greatly be reduced.

The grape cluster in the Figure 8b was mostly occluded and only the bottom part of the grape cluster was ultimately detected. In this case, the obtained information would hardly meet the requirement of the harvesting robots. To know how much the pixels recognition accuracy (PRA) can meet the requirement of the harvesting robots, 22 grape clusters with different occlusion degrees in the collected images were taken as the test cases, the PRA of these grape clusters were calculated by the following Equation (15):

(15) $$\mathrm{PRA}=\frac{{\mathrm{Region}}_{\mathrm{manual}}\cap {\mathrm{Region}}_{\mathrm{algorithm}}}{{\mathrm{Region}}_{\mathrm{manual}}}\times 100\%$$

where ${\mathrm{Region}}_{\mathrm{manual}}$ is the pixels region of grape clusters that were extracted manually, and ${\mathrm{Region}}_{\mathrm{algorithm}}$ is the pixels region of grape clusters acquired by the algorithm. The testing results indicated that the grape clusters could be well detected when 75% or more of grape pixels region were correctly classified.

**Figure 9:**
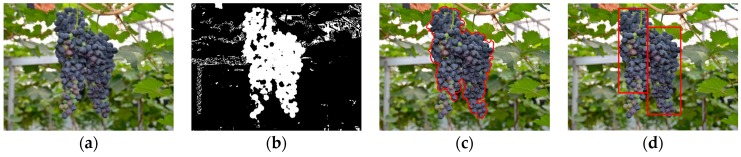
Detection process of one of the multiple overlapping grape clusters (**a**) Original image; (**b**) Initial segmentation image by the developed classifier; (**c**) The contour of the detected grape clusters; and (**d**) Detection result.

When some unripe grape berries appeared among the ripe grape clusters or multiple grape clusters overlapped, false or failure identification would occur, and it would be difficult to accurately detect the grape clusters. As it can be seen in Figure 9, some unripe berries whose color was green and three overlapping grape clusters could be found in Figure 9a, and from the segmentation result in Figure 9b we can see that those unripe berries at the bottom of the left grape clusters were not identified, so that final detection results in Figure 9c were not ideal. At last, only the right grape cluster was correctly detected, the left grape clusters were not accurately detected.

**Figure 10:**
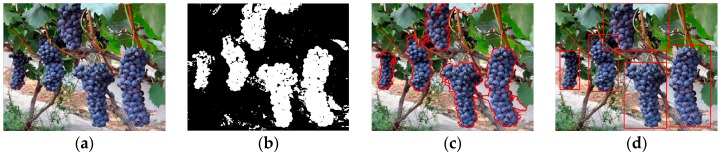
Detection process of the Cabernet Sauvignon grape clusters (**a**) Original image; (**b**) Initial segmentation image by the developed classifier; (**c**) The contour of the detected grape clusters; and (**d**) Detection result.

In addition, in order to test the applicability of the developed approach for other black varieties besides the tested ‘Summer Black’ grape variety, the ‘Cabernet Sauvignon’ whose color was similar to the ‘Summer Black’ was also tested. As it can be seen in Figure 10, the grape clusters in the images were finally detected, and only a small number of ground and reflective leaves pixels were misclassified into the grape. However, it could be seen that the developed approach was sensitive to the color information and the training samples had important influence on the final detection results.

### 3.4. Comparing the Proposed Approach with Other Approach

To make comparison with the proposed approach, the recent grape bunch detection method that was presented in the paper [18] was employed to test. Firstly, 14 grape clusters in the collected vineyard images were randomly chosen as the testing objects, and then all pixels region of the chosen grape clusters were extracted manually to properly evaluate the results. Secondly, the grape clusters were detected by using the two approaches, and then the intersection between the pixels region extracted by manual and the pixels region detected by the algorithms were calculated out. Finally, the pixel recognition accuracy (PRA) was computed by the previous Equation (15).

**Figure 11:**
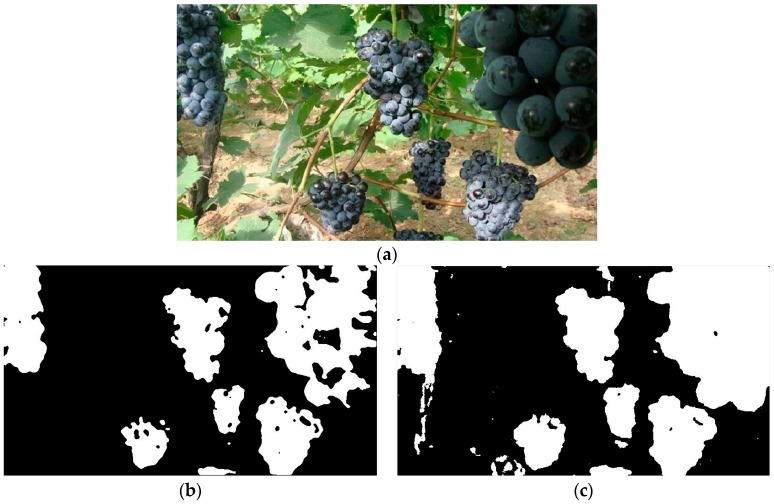

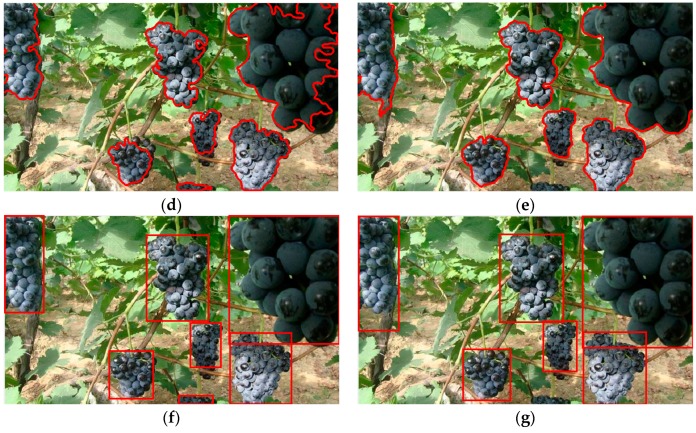
The comparison of the detection process between the method [18] and the proposed method, (**a**) Original image; (**b**) Initial segmentation image by the method [18]; (**c**) Initial segmentation image by the proposed method; (**d**) The detected contour of the grape clusters by the method [18]; (**e**) The detected contour of the grape clusters by the proposed method; (**f**) The detection result by using the method [18]; and (**g**) The detection result by using the proposed method.

The RPA comparison between the method [18] and the proposed method were listed in Table 4. From the table we could know that the average PRA of the proposed method was higher than with the method described in [18]. Figure 11 shows the detection process on one of the collected vineyard images by using the two methods. It is clear that the detection results of the proposed method were better those of [18], and the over-segmentation was more serious in [18] than with the proposed method, which can be seen from the right-up grape cluster. In [18], the key step of grape cluster image segmentation was mainly based on the H and V components of the HSV color space, after obtaining initial bunch areas, and the false detected bunches were eliminated by combining texture information and the use of a support vector machine. However, by simply relying on one of the color spaces, it was difficult to smoothly extract the initial bunch areas from the complex vineyard environment with changing illumination.

### 3.5. The Interactive Performance of the Developed Approach

**Figure 12:**
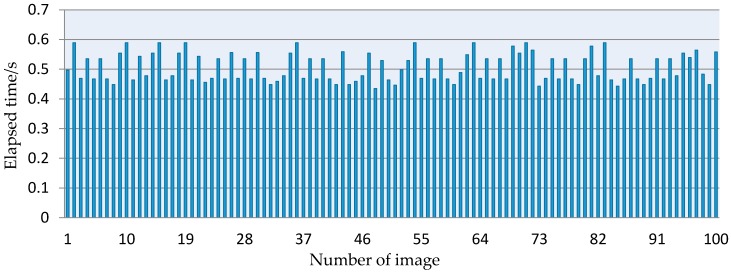
The elapsed time of 100 captured images in the vineyard.

The detection speed on the targets, which determines the harvesting efficiency of robots, is very crucial for robotic harvesting. To determine whether the developed method could be used in harvesting robots, the elapsed time of the whole algorithm was computed using the clock function in Matlab, and the experiments were conducted on the 100 images that were randomly chosen from the captured images. The elapsed time of these images was showed in Figure 12. The result indicated that the elapsed time of every image was less than 0.59 s, and the overall processing time varied approximately from 0.43 s to 0.59 s.

## 4. Discussion

To detect the grape clusters in the vineyard, four color-based linear weak classifiers were firstly constructed in this study, which have their own advantages and disadvantages. To obtain a robust classifier, a strong classifier was constructed by assembling the four weak classifiers using the AdaBoost framework, which takes advantages of the four weak classifiers and makes up for their insufficiency to achieve the desired results. The grape clusters in the vineyard under different lighting conditions were tested by combining the strong classifier and the contour enclosing rectangle extraction method. The experimental results demonstrated that the constructed strong classifier could well identify the grape clusters from the backgrounds (e.g., leaves, branches and sky, etc.). From the experiments performed on 900 testing samples we can know that the classification accuracy reached up to 96.56%, and the experiment on 200 vineyard images showed that the success rate of the developed detection approach was 93.74%. The performance tests indicated that the approach could partly account for the influence of the complex background features such as the weather conditions, leaves and changing illumination. From the interactive performance of the developed approach in the tests, the processing time of each image was less than 0.59 s, so it could meet the requirements of harvesting robots. This demonstrated performance indicates that the approach could successfully and robustly detect grape clusters in the vineyard, and it could be embedded into the vision system of harvesting robots to help them grasp grape clusters accurately [39].

However, there were still some shortcomings of the developed approach. First, when the illumination intensity in the vineyard changes dramatically, the color of the collected images will inevitably change. Although the training samples of the constructed classifier have included three kinds different illumination, it couldn’t cover all illuminations, so a part of the pixels may be wrongly classified at times. The morphological denoising processing could remove some noises, but false negatives may still occur, and then the developed approach would not work. In such a situation, a further performance detection of grape berries in the enclosing rectangle of targets by using the Hough circle detection method, which can identify the pixels region of targets whether or not be grape clusters, may be a good solution. Second, when most of the grape clusters was overlapped or occluded, some grape clusters may not be detected successfully. The detection of overlapping or occluded fruits has been a difficult issue for harvesting robots, especially the grape clusters whose contour were irregular. Therefore, further research is needed to improve the detection accuracy of overlapped or occluded grape clusters in the vineyard. Moreover, because the developed approach relied on the color information of grape clusters, it can only be well utilized for the kind of grape which was chosen as the training samples of the proposed classifiers, so if we want to use the developed method with other grape varieties whose color is very different, the training samples of the classifiers have to be reprepared, and the effective color components of the grape need be redetermined too. In addition, the color of unripe grape berries is usually different from the ripe ones, so when the ripe grape clusters are mingled with a few unripe berries, the detection results would be affected.

Based on the discussions above, we can know that the developed approach can be used for vineyard harvesting robotd. However, additional research is still needed to improve the detection rate and accommodate more varied unstructured environments.

## 5. Conclusions and Future Work

To comply with the development of precision agriculture/viticulture, an approach to detect ripe grape clusters in the vineyard for the harvesting robot was developed in this paper. At the beginning of the approach, the effective color components of H, Cb, b*, R, G and B which could well distinguish the summer black grape from the background were extracted, based on which the corresponding linear classifiers were built. To identify the grape clusters smoothly, the strong and robust classifier was constructed by combining the AdaBoost algorithm and the linear weak classifier. Then, all pixels of images were classified by the strong classifier and the targets of grape clusters were finally extracted by the enclosing rectangle method. Two experiments were implemented by using the images from vineyard scene and the results were quantitatively assessed and compared with other recent classification methods. The main results are as follows:

(1) The strong classifier was able to automatically distinguish the grapes from background, and the accuracy of the classifications can reach up to 96.56%, which was higher than with any weak classifier.
(2) The success rate of the proposed detection algorithm was 93.74%, which was superior to other weak classifiers.
(3) The interactive performance of the proposed detection algorithm was investigated, and the elapsed time of every image was less than 0.59 s, which can meet the requirements of harvesting robots.

In conclusion, the developed approach can effectively detect the harvesting targets in the complex vineyard environment. However, the accurate detection of multiple overlapped and adjoining grape clusters is still an issue that needs to be solved, and will require further research. In addition, the developed approach only can be utilized well for the kind of grape which was choosen as the training samples of the proposed classifiers. To solve these tricky problems, in future research we will consider modeling the whole scene by using a depth camera sensor to get depth maps of the scenes from different angles of view, and then combine the color image segmentation and intelligent methods to detect the overlapping or adjoining targets.

## Figures and Tables

**Table 1:** The error rate of weak classifiers and their weights in the final strong classifier.

Classifiers	Classifiers of Equation	Errors/$\varepsilon_{t}$	Weight of Weak Classifiers/$\alpha_{t}$
$h_{1}\left(x_{i}\right)$	*H*($x_{i}$) − 0.57 = 0	0.044	1.537
$h_{2}\left(x_{i}\right)$	*Cb*($x_{i}$) − 0.86 = 0	0.119	1.001
$h_{3}\left(x_{i}\right)$	0.31 − *Lab_b*($x_{i}$) = 0	0.225	0.620
$h_{4}\left(x_{i}\right)$	10×*B*($x_{i}$) − 6.16×*R*($x_{i}$) − 1.3 = 0	0.341	0.331
$H\left(x_{i}\right)$	$sign\left\{1.537\times h_{1}\left(x_{i}\right)+1.001\times h_{2}\left(x_{i}\right)+0.620\times h_{3}\left(x_{i}\right)+0.331\times h_{4}\left(x_{i}\right)\right\}$		

**Table 2:** Comparison of the classifiers accuracy.

Classifiers	Actual Categories	Samples Number	Classified Categories		TPR/%	FPR/%
			Grape	Background		
$h_{1}\left(x_{i}\right)$	Grape	300	276	24	93.67	6.33
	Background	600	33	567		
$h_{2}\left(x_{i}\right)$	Grape	300	231	69	90.22	9.78
	Background	600	19	581		
$h_{3}\left(x_{i}\right)$	Grape	300	259	41	81.33	18.67
	Background	600	127	473		
$h_{4}\left(x_{i}\right)$	Grape	300	278	22	83.56	16.44
	Background	600	126	474		
$H\left(x_{i}\right)$	Grape	300	282	18	96.56	3.44
	Background	600	13	587		

**Table 3:** The detection results of the proposed method.

Lighting Conditions	Grape Clusters	True Positives		False Negatives		False Positives	
		Amount	TPR/%	Amount	FNR/%	Amount	FPR/%
Sunny frontlight	136	128	94.12	5	3.76	8	5.88
Sunny overshadow	129	118	91.47	6	4.84	11	8.53
Overcast lighting	182	173	95.05	8	4.42	9	4.95
Total	447	419	93.74	19	4.34	28	6.26

**Table 4:** The PFR comparison of the proposed method and the method [18].

Sequence Number of Grape Clusters	The RPA of Paper [18]/%	The RPA of the Proposed Method/%
1	91.27	93.43
2	86.82	92.86
3	88.76	93.73
4	90.57	89.23
5	91.75	89.75
6	90.26	91.26
7	92.27	95.23
8	89.78	88.95
9	86.53	89.26
10	87.36	91.23
11	89.26	93.69
12	93.45	92.26
13	88.36	87.63
14	91.59	94.45
**Average value**	**89.86**	**91.49**
